# SNPs in LncRNA genes are associated with non‐small cell lung cancer in a Chinese population

**DOI:** 10.1002/jcla.22858

**Published:** 2019-04-13

**Authors:** Ruoyang Wang, Nannan Feng, Yu Wang, Sumeng Gao, Fangfang Zhang, Ying Qian, Ming Gao, Herbert Yu, Baosen Zhou, Biyun Qian

**Affiliations:** ^1^ School of Public Health, Shanghai Jiaotong University School of Medicine, Hongqiao International Institute of Medicine Shanghai Tongren Hospital Shanghai China; ^2^ Key Laboratory of Cancer Prevention and Therapy Tianjin Medical University Cancer Institute and Hospital Tianjin China; ^3^ Cancer Epidemiology Program University of Hawaii Cancer Center Honolulu Hawaii; ^4^ Department of Epidemiology, School of Public Health China Medical University Shenyang China

**Keywords:** long non‐coding RNA, non‐small cell lung cancer, single nucleotide polymorphism, survival, susceptibility

## Abstract

**Background:**

It has indicated that single nuclear polymorphisms (SNPs) in the regions encoding non‐coding transcripts are associated with lung cancer susceptibility. In a previous microarray study, we identified 13 differentially expressed long non‐coding RNAs (lncRNAs) in non‐small cell lung cancer (NSCLC) and associations of SNPs in these lncRNA genes with lung cancer were unknown. We conducted a case‐control study to address this issue.

**Methods:**

Using the TaqMan method, we genotyped 17 SNPs located in the 13 lncRNA genes in 1294 cases with NSCLC and 1729 healthy controls. Unconditional logistic regression and Cox proportional hazards regression were used to analyze the associations of these SNPs with NSCLC risk and patient survival, respectively. These analyses were also repeated in subgroups of cases and controls stratified by gender, age group, smoking status, disease stage, and histological type.

**Results:**

We identified three SNPs associated with NSCLC risk. For SNP rs498238, CC genotype was associated with lower risk compared to TT genotype (adjusted OR = 0.33, 95%CI: 0.11‐0.97, *P* = 0.043). For rs16901995, CT/TT genotypes were associated with lower risk compared to CC genotype in non‐smokers (adjusted OR = 0.78, 95%CI: 0.62‐0.98, *P* = 0.035). Variant genotypes in rs219741 were associated with NSCLC risk in young patients, and the adjusted OR was 1.47 (95%CI: 1.03‐2.10, *P* = 0.033) when compared to the wild genotype. No SNPs were found to be associated with patient overall survival in the study.

**Conclusion:**

The study suggests that some genetic polymorphisms in the lncRNA genes may influence the risk of NSCLC among Chinese.

## 1 INTRODUCTION

Lung cancer is the leading cause of cancer death worldwide, and 22% and 13.8% of cancer deaths in 2018 were estimated to be caused by lung cancer in men and women, respectively.^1^ The corresponding percentages in China were 28% and 23% in 2012,^2^ making it the most common cause of cancer death. Lung cancer is classified into two main categories: non‐small cell lung cancer (NSCLC), accounting for approximately 80% of all lung cancer cases, and small‐cell lung cancer (SCLC).^3^ Although tobacco smoking is the major risk factor,^4^ the etiology of lung cancer is multifactorial, including inherited genetic characteristics, such as single nucleotide polymorphisms (SNPs),^5^ which explains individual's susceptibility to the development of lung cancer. During the past decade, genome‐wide association studies (GWAS) have identified many common SNPs associated with the risk and outcome of lung cancer. However, heritability analysis indicated that the identified genetic loci could explain only a small fraction of lung cancer susceptibility.^6^ Additional efforts are needed to search for more lung cancer‐related genetic factors, especially those rare variants and loci in non‐coding regions.

Long non‐coding RNAs (lncRNAs) are a class of RNA transcripts with more than 200 nucleotides in length and without translational capability. LncRNAs have been found to have diverse biological functions, some of which are involved in various tumorigenic processes.^7^ A number of dysregulated lncRNAs have also been demonstrated to be potential diagnostic or prognostic biomarkers for lung cancer, such as metastasis associated in lung adenocarcinoma transcript 1 (*MALAT1*)^8^ and HOX antisense intergenic RNA (*HOTAIR*)^9^ which are overexpressed in NSCLC and recognized as onco‐lncRNAs. In contrast, maternally expressed gene 3 (*MEG3*),^10^ taurine‐upregulated gene 1 *(TUG1*),^11^ and BRAF‐activated non‐protein coding RNA (*BANCR*)^12^ which are downregulated in NSCLC are considered as tumor suppressors. These dysregulated lncRNAs are found to be involved in regulation of cell growth, proliferation, migration, and invasion.

Evidence also indicates that SNPs in the lncRNA genes affected tumorigenic process and chemotherapy response. Gong et al^13^ found that SNPs in *HOTTIP*, *H19,* and *CCAT2* were associated with lung cancer risk, and SNPs in *MALAT1*, *H19*, *CCAT2*, *HOTAIR,* and *ANRIL* were related to lung cancer patients’ response to platinum‐based chemotherapy. Yuan et al^14^ conducted a meta‐analysis of eight GWAS on subjects with European ancestry and discovered rs114020893 in the lncRNA *NEXN‐AS1* associated with lung cancer risk. This SNP's influence on lung cancer susceptibility may be achieved through its genotype‐specific secondary structure stability. Hu et al^15^ reported a SNP in *CASC8* associated with both lung cancer risk and chemotherapy response and toxicity.

Findings from the above studies indicate that identifying SNPs in the lncRNA genes associated with lung cancer may help to elucidate the biological mechanisms of lncRNAs in lung cancer. Currently, our knowledge on lncRNA's involvement in lung cancer is still limited; more studies are needed to discover SNPs in lncRNAs which are associated with lung cancer risk or outcome. Based on the findings of our previous study on lncRNAs in NSCLC,^16^ we conducted a case‐control study on SNPs of the lncRNAs which showed different expression between tumor and matched adjacent normal tissues. In this study, we analyzed the association of lung cancer with 17 SNPs in 13 selected lncRNAs. We also investigated these SNPs in relation to lung cancer survival. Results of our association study are described in this report.

## 2 MATERIALS AND METHODS

### 2.1 Study subjects

The case‐control study included 1294 NSCLC cases and 1729 healthy controls who were recruited between April 2011 and July 2015 from the China Medical University. The cases were newly diagnosed patients with histologically confirmed primary NSCLC who had no previous diagnosis of cancer or treatment of radiotherapy and chemotherapy. The cases were followed after surgery until August 2017 through clinical visits and regular telephone contacts. The control subjects were identified and enrolled in the study from the same or nearby communities where the cases resided. The controls had no medical history of cancer at the time of case diagnosis. All the study subjects were genetically unrelated Chinese with Han ethnicity. The study was approved by the Medical Ethics Committees of Human Studies at China Medical University. Written informed consents were obtained from all the subjects.

### 2.2 SNP selection and genotyping

In our previous study,^16^ we found 153 lncRNAs, which had significant differences in expression (fold change >2) between tumor and matched adjacent tissues. Based on the list, we searched NCBI dbSNP (http://www.ncbi.nlm.nih.gov/), HapMap (http://www.hapmap.org), and lncRNASNP (http://bioinfo.life.hust.edu.cn/lncRNASNP/) and identified 3765 SNPs. Considering that polymorphisms in the non‐coding regions may affect the binding of other transcripts such as microRNAs,^17^ we selected SNPs located in the binding sites which may alter the binding affinity of lncRNAs to other molecules. The following selection criteria were established to choose SNPs for genotyping: (a) minor allele frequency (MAF) reported in HapMap ≥5% in Chinese Han, Beijing (CHB); (b) located in the regulatory region of genes; and (c) affecting the binding with microRNAs. Following the criteria, we selected 17 SNPs for study (Table 1).

**Table 1:** Information on 17 SNPs in the 13 lncRNA genes

Rs number	Gene	Locus	Location	Base change	MAF in controls	HWE *P*
rs10889184	LINC01748	1p32.1	60540378	G/A	0.45	0.622
rs3113503	LINC00607/LINC01614	2q35	215719150	G/C	0.33	0.849
rs498238	LINC01833	2p21	44921691	C/T	0.12	0.624
rs496467	LINC01833	2p21	44921864	A/G	0.49	0.702
rs13431201	LINC01833	2p21	44922015	C/G	0.06	0.440
rs1992825	LINC01833	2p21	44923139	G/C	0.32	0.242
rs517055	LINC01833	2p21	44923338	A/T	0.49	0.573
rs1466099	RNF144A‐AS1	2p25.2	6917071	G/A	0.26	0.819
rs62288095	LINC00887	3q29	194303359	C/A	0.11	0.512
rs6830064	LINC02466	4q28.2	129725387	T/G	0.18	0.694
rs7678341	lnc‐RCHY1‐3:1	4q13.3	75269312	G/A	0.23	0.541
rs16901995	lnc‐NDUFS6‐5:5	5p15.33	1933867	C/T	0.42	0.978
rs4077205	LOC100128340	5q35.3	177957648	A/G	0.70	0.107
rs35132843	CASC21/CASC8	8q24.21	127289874	T/G	0.37	0.087
rs10734387	BBOX1‐AS1	11p14.2	27151108	C/T	0.30	0.638
rs1867299	HOXC13‐AS	12q13.13	53936191	T/C	0.18	0.135
rs219741	LOC105369301	21q22.13	36480738	G/A	0.10	0.716

HWE, Hardy‐Weinberg equilibrium; MAF, Minor allele frequency.

Our genotyping method has been described elsewhere.^18^ In brief, genomic DNA in peripheral blood leukocytes was extracted from cases and controls using the standard phenol‐chloroform method. SNP genotyping was determined by the TaqMan assay using the ABI 7900 FAST real‐time polymerase chain reaction (PCR) system (Thermo Fisher Scientific, Waltham, MA, USA). All primers and probes were purchased from Thermo Fisher Scientific. Ten percent of the DNA samples were randomly selected for replication, and the results of the repeats were in complete concordance.

### 2.3 Statistical analysis

Distributions of subject characteristics and genetic polymorphisms were compared between cases and controls using the chi‐square test. Student *t* test was used for comparison of continues variables between groups. Hardy‐Weinberg equilibrium was calculated for each SNP in the control subjects. In order to balance the distributions of age and gender in case and control groups, propensity score matching (PSM) analysis was conducted. Associations between SNPs and NSCLC risk were analyzed using the unconditional logistic regression model. Odds ratios (OR) and 95% confidence interval (CI) were calculated in the regression model, and the analyses were adjusted for confounding factors (age, gender, and smoking status). Subgroup analyses were also performed for each polymorphism to assess potential gene‐environment interaction or joint effect. Survival time was defined as the time interval from the date of NSCLC diagnosis to the date of death or end of follow‐up. Median survival time (MST) was the time point when 50% of the patients were dead. Kaplan‐Meier survival analysis and log‐rank test were used to compare differences in survival time by SNP genotypes. Associations between SNPs and overall survival were analyzed using the Cox proportional hazards regression model in which hazard ratios (HR) and 95%CI were estimated. *P* values reported were two‐tailed, and *P *< 0.05 was considered statistically significant. All data analyses were performed using the SPSS software version 19.0 (IBM, Armonk, NY, USA). We also selected the NCBI data sets, GSE19804 and GSE18842, for analysis of gene expression. The scatter plots were generated using the GraphPad Prism 6.0 software (GraphPad Software, San Diego, CA, USA).

## 3 RESULTS

### 3.1 Study population

The demographic characteristics of the initial 1294 cases and 1729 controls were summarized in Table S1. In order to balance the age and gender differences between cases and controls, we conducted PSM. First, we deleted subjects with missing values in gender and age, which left us with 1169 NSCLC cases and 1354 controls. Then, a propensity score (PS) was constructed to quantify each subject's gender and age. The cases were later matched to controls by PS. After PSM, we obtained well‐balanced distributions of demographic characteristics between cases and controls (Table 2). The age (*P* = 0.310) and gender (*P* = 0.326) were no longer significantly different. There were more smokers in cases than in controls (48.07% vs 25.37%).

**Table 2:** Distribution of the selected characteristics in cases and controls after PSM

Variables	N (%)		*P* value*
	Case (n = 1169)	Control (n = 1005)	
Gender	1169 (100%)	1005 (100%)	0.326
Male	579 (49.53%)	519 (51.64%)	
Female	590 (50.47%)	486 (48.36%)	
Age	1169 (100%)	1005 (100%)	0.310
<60	584 (49.96%)	524 (52.14%)	
≥60	585 (50.04%)	481 (47.86%)	
Smoking status^a^	1165 (100%)	1001 (100%)	<0.001
Non‐smoker	605 (51.93%)	747 (74.63%)	
Ever‐smoker	560 (48.07%)	254 (25.37%)	

a Due to the missing values, the numbers of cases and controls were less than 1169 and 1005, respectively.

* Two‐side chi‐squared test.

### 3.2 Associations of SNPs and NSCLC risk

Allele distributions of the 17 SNPs selected for study were all in Hardy‐Weinberg equilibrium in the control group (*P* > 0.05, Table 1). After PSM, genotype distributions of the 17 SNPs and their associations with NSCLC risk in different inheritance models (dominant, recessive, and additive) are shown in Tables S2 and 3. Potential gene‐environment interaction was assessed for each polymorphism in the initial study population stratified by the environmental factor of interest (Table 4). Significant associations with NSCLC were suggested for three SNPs, including rs498238, rs16901995, and rs219741.

**Table 3:** Associations between selected SNPs and NSCLC risk after PSM

Genotypes	N (%)		*P* value*	Crude OR (95%CI)	Adjusted OR (95%CI)*, a
	Case	Control			
**rs3113503 (G>C)**	1012 (100%)	979 (100%)	0.050		
GG	432 (42.69%)	448 (45.76%)		1.00	1.00
GC	489 (48.32%)	423 (43.21%)		1.20 (1.00‐1.44)	**1.22 (1.01‐1.49)**
CC	91 (8.99%)	108 (11.03%)		0.87 (0.64‐1.19)	0.81 (0.59‐1.13)
Dominant model	1012 (100%)	979 (100%)	0.167		
GG	432 (42.69%)	448 (45.76%)		1.00	1.00
GC + CC	580 (57.31%)	531 (54.24%)		1.13 (0.95‐1.35)	1.14 (0.94‐1.37)
Recessive model	1012 (100%)	979 (100%)	0.129		
GG + GC	921 (91.01%)	871 (88.97%)		1.00	1.00
CC	91 (8.99%)	108 (11.03%)		0.80 (0.59‐1.07)	**0.74 (0.54‐1.00)**
**rs498238 (C>T)**	1005 (100%)	961 (100%)	0.188		
CC	791 (78.71%)	745 (77.52%)		1.00	1.00
CT	209 (20.80%)	204 (21.23%)		0.97 (0.78‐1.20)	0.97 (0.77‐1.22)
TT	5 (0.50%)	12 (1.25%)		0.39 (0.14‐1.12)	**0.33 (0.11‐0.97)**
Dominant model	1005 (100%)	961 (100%)	0.526		
CC	791 (78.71%)	745 (77.52%)		1.00	1.00
CT + TT	214 (21.29%)	216 (22.48%)		0.93 (0.75‐1.16)	0.93 (0.74‐1.16)
Recessive model	1005 (100%)	961 (100%)	0.072		
CC + CT	1000 (99.50%)	949 (98.75%)		1.00	1.00
TT	5 (0.50%)	12 (1.25%)		0.40 (0.14‐1.13)	**0.33 (0.11‐0.97)**
**rs16901995 (C>T)**	1096 (100%)	984 (100%)	0.413		
CC	380 (34.67%)	322 (32.72%)		1.00	1.00
CT	532 (48.54%)	477 (48.48%)		0.95 (0.78‐1.15)	0.94 (0.77‐1.15)
TT	184 (16.79%)	185 (18.80%)		0.84 (0.66‐1.09)	0.78 (0.59‐1.01)
Dominant model	1096 (100%)	984 (100%)	0.348		
CC	380 (34.67%)	322 (32.72%)		1.00	1.00
CT + TT	716 (65.33%)	662 (67.28%)		0.92 (0.76‐1.10)	0.89 (0.74‐1.08)
Recessive model	1096 (100%)	984 (100%)	0.230		
CC + CT	912 (83.21%)	799 (81.20%)		1.00	1.00
TT	184 (16.79%)	185 (18.80%)		0.87 (0.70‐1.09)	0.80 (0.63‐1.02)
**rs219741 (G>A)**	1056 (100%)	955 (100%)	0.325		
GG	813 (76.99%)	753 (78.85%)		1.00	1.00
GA	235 (22.25%)	191 (20.00%)		1.14 (0.92‐1.41)	1.08 (0.86‐1.35)
AA	8 (0.76%)	11 (1.15%)		0.67 (0.27‐1.68)	0.60 (0.23‐1.56)
Dominant model	1056 (100%)	955 (100%)	0.316		
GG	813 (76.99%)	753 (78.85%)		1.00	1.00
GA + AA	243 (23.01%)	202 (21.15%)		1.11 (0.90‐1.38)	1.05 (0.84‐1.31)
Recessive model	1056 (100%)	955 (100%)	0.361		
GG + GA	1048 (99.24%)	944 (98.85%)		1.00	1.00
AA	8 (0.76%)	11 (1.15%)		0.66 (0.26‐1.64)	0.59 (0.23‐1.53)

Bold OR values indicated *P* < 0.05.

a Adjusted for age, gender, and smoking status.

* Two‐side chi‐squared test.

**Table 4:** Associations between SNPs and NSCLC risk stratified by selected variables

Genetic Variant	Variables	Genotypes (Cases/Controls)		*P* value^a^	Dominant model (AB + BB)/AA OR (95%CI)^a^
		AA*, b	AB + BB*, b		
rs3113503	Gender				
	Male	231/235	280/338	0.370	0.88 (0.67‐1.16)
	Female	202/463	301/511	0.054	1.29 (1.00‐1.66)
	Age				
	<60	225/390	273/458	0.877	0.98 (0.74‐1.30)
	≥60	207/204	307/269	0.196	1.20 (0.91‐1.57)
	Smoking status				
	Non‐smoker	221/641	302/765	0.373	1.11 (0.88‐1.39)
	Ever‐smoker	215/120	276/148	0.589	1.09 (0.80‐1.48)
rs498238	Gender				
	Male	391/426	114/139	0.662	0.93 (0.67‐1.29)
	Female	402/754	100/192	0.711	0.94 (0.69‐1.29)
	Age				
	<60	390/648	107/171	0.518	0.89 (0.63‐1.26)
	≥60	401/362	107/108	0.623	0.92 (0.67‐1.27)
	Smoking status				
	Non‐smoker	410/1081	107/295	0.936	0.99 (0.75‐1.31)
	Ever‐smoker	385/200	105/64	0.334	0.84 (0.58‐1.20)
rs16901995	Gender				
	Male	177/180	371/396	0.918	0.99 (0.74‐1.31)
	Female	204/333	346/648	0.202	0.85 (0.66‐1.09)
	Age				
	<60	195/278	346/577	0.076	0.77 (0.58‐1.03)
	≥60	185/154	370/321	0.698	1.06 (0.80‐1.40)
	Smoking status				
	Non‐smoker	212/469	354/950	**0.035**	**0.78 (0.62‐0.98)**
	Ever‐smoker	168/89	364/180	0.679	1.07 (0.78‐1.48)
rs219741	Gender				
	Male	407/456	127/109	0.617	1.09 (0.79‐1.50)
	Female	408/747	116/168	0.651	1.07 (0.79‐1.46)
	Age				
	<60	405/676	119/125	**0.033**	**1.47 (1.03‐2.10)**
	≥60	408/351	124/119	0.433	0.88 (0.65‐1.20)
	Smoking status				
	Non‐smoker	420/1096	121/238	0.400	1.13 (0.86‐1.48)
	Ever‐smoker	395/210	122/59	0.933	1.02 (0.71‐1.45)

Bold OR values indicated *P* < 0.05.

a Adjusted for age, gender, and smoking status when properly.

b A stands for major allele and B stands for minor allele.

For SNP rs498238, individuals with the TT homozygous genotype had a lower risk of NSCLC compared to those with the CC homozygous genotype after age, gender, and smoking status were adjusted in the analysis (adjusted OR = 0.33, 95%CI: 0.11‐0.97, *P* = 0.043; Table 3). The association between rs498238 and NSCLC mainly came from the recessive model, and no significant association was seen in the dominant model.

SNP rs16901995 was not associated with NSCLC in overall analysis, but in the stratified analysis it was shown that in non‐smokers, individuals with CT or TT genotypes had a reduced risk for NSCLC compared to those with CC genotype (adjusted OR = 0.78, 95%CI: 0.62‐0.98, *P* = 0.035; Table 4). Similarly, when analyzing the relationship in subgroups, we found that SNP rs219741 was associated with increased risk of NSCLC among younger subjects (age < 60 years). The adjusted OR was 1.47, and 95%CI was between 1.03 and 2.10 (*P* = 0.033).

SNP rs3113503 showed controversial results. Individuals with GC genotype had an increased risk compared to those with wild GG genotype (adjusted OR = 1.22, 95%CI: 1.01‐1.49, *P* = 0.035). But subjects with CC genotype had a reduced risk in a recessive model (adjusted OR = 0.74, 95%CI: 0.54‐1.00, *P* = 0.050). There was no significant difference in the dominant model, nor in stratified analyses.

### 3.3 Associations of SNPs and NSCLC outcome

Patient characteristics and clinical features are shown in Table S3. Survival analysis was performed to assess the genotypes of the four selected SNPs in association with the NSCLC outcome (Table 5). The analysis showed no significant associations between these genotypes and NSCLC overall survival before or after adjustment for age, gender, smoking status, disease stage, and histology type. To further investigate the association of SNPs with NSCLC survival in patients with different clinical characteristics, we conducted stratification analyses in the dominant model (Table S4). The results showed that only in patients with lung adenosquamous carcinoma (ASC), rs219741 was associated with survival. However, the sample size (deaths/patients: 19/23 vs 5/6, in GG vs GA + AA genotypes, respectively) was too small to draw a conclusion.

**Table 5:** Associations between SNPs and NSCLC survival

Genotypes	Patients	Deaths	MST (mo) (95%CI)	Log‐rank *P*	HR (95%CI)	HR (95%CI)^a^
**rs3113503**	746	457		0.955		
GG	326	198	29.43 (23.45‐35.42)		1.00	1.00
CG	351	215	29.33 (23.60‐35.06)		1.03 (0.85‐1.25)	1.03 (0.84‐1.26)
CC	69	44	29.40 (21.22‐37.58)		1.02 (0.74‐1.42)	1.05 (0.74‐1.48)
Dominant model				0.764		
GG	326	198	29.43 (23.45‐35.42)		1.00	1.00
CG + CC	420	259	29.37 (24.32‐34.42)		1.03 (0.86‐1.24)	1.03 (0.85‐1.25)
Recessive model				0.963		
GG + CG	677	413	29.33 (25.02‐33.64)		1.00	1.00
CC	69	44	29.40 (21.22‐37.58)		1.01 (0.74‐1.38)	1.03 (0.74‐1.44)
**rs219741**	777	467		0.500		
GG	610	359	31.97 (26.69‐37.25)		1.00	1.00
AG	161	104	28.80 (22.88‐34.73)		1.14 (0.92‐1.42)	1.10 (0.88‐1.39)
AA	6	4	29.37 (22.73‐36.00)		1.02 (0.38‐2.74)	1.11 (0.41‐2.99)
Dominant model				0.248		
GG	610	359	31.97 (26.69‐37.25)		1.00	1.00
AG + AA	168	109	29.27 (25.06‐33.48)		1.14 (0.92‐1.41)	1.11 (0.88‐1.38)
Recessive model				0.992		
GG + AG	771	463	31.00 (27.03‐34.97)		1.00	1.00
AA	6	4	29.37 (22.73‐36.00)		1.00 (0.37‐2.66)	1.09 (0.41‐2.93)
**rs498238**	737	448		0.902		
CC	574	353	29.40 (24.44‐34.36)		1.00	1.00
TC	158	91	32.33 (25.43‐39.24)		0.98 (0.77‐1.23)	1.02 (0.80‐1.30)
TT	5	4	28.30 (6.68‐49.92)		1.22 (0.45‐3.26)	0.83 (0.31‐2.24)
Dominant model				0.884		
CC	574	353	29.40 (24.44‐34.36)		1.00	1.00
CC + TC	163	95	32.33 (25.64‐39.03)		0.98 (0.78‐1.23)	1.01 (0.79‐1.28)
Recessive model				0.689		
CC + TC	732	444	29.97 (26.02‐33.91)		1.00	1.00
TT	5	4	28.30 (6.68‐49.92)		1.22 (0.46‐3.27)	0.83 (0.31‐2.23)
**rs16901995**	810	499		0.690		
CC	294	182	27.23 (23.06‐31.40)		1.00	1.00
CT	386	239	33.00 (26.27‐39.73)		0.94 (0.77‐1.14)	0.98 (0.80‐1.21)
TT	130	78	29.97 (22.49‐37.44)		0.90 (0.70‐1.17)	0.96 (0.73‐1.28)
Dominant model				0.421		
CC	294	182	27.23 (23.06‐31.40)		1.00	1.00
CT + TT	516	317	32.53 (27.80‐37.27)		0.93 (0.77‐1.11)	0.98 (0.81‐1.19)
Recessive model				0.580		
CC + CT	680	421	28.80 (24.85‐32.75)		1.00	1.00
TT	130	78	29.97 (22.49‐37.44)		0.93 (0.73‐1.19)	0.97 (0.75‐1.26）

a Adjusted for age, gender, smoking status, disease stage, and histology type.

## 4 DISCUSSION

In this study, we evaluated 17 SNPs in 13 lncRNAs with regard to their associations with NSCLC risk and survival. We found that NSCLC risk was significantly associated with SNP rs3113503, rs498238, rs16901995, and rs219741. These SNPs are located in different lncRNA genes and appeared to have different associations with NSCLC. While SNP rs219741 was associated with an increased risk in younger population, SNP rs498238 and rs16901995 were linked to a reduced risk of NSCLC. SNP rs3113503 had a conflicting relationship with NSCLC risk.

Although the biological implications of these SNPs in the lncRNA genes are unknown, our understanding of lncRNA's involvement in cancer is rapidly expanding in recent years. The biological function of lncRNA largely depends on their subcellular localization. In cytoplasm, lncRNAs behave like competitive endogenous RNA to bind mRNAs, suppressing translation or degradation of targeted mRNAs. When in nucleus, lncRNAs serve as scaffold to form, for example, a chromatin modification complex, or act as decoy to suppress the function of other transcripts, such as microRNAs. Some lncRNAs tether transcription factors to gene promoters.^7^ Recently, lncRNAs are found to contain codes for functional micropeptides based on small‐ORFs (Open Reading Frames).^19^ LncRNAs may also play roles in intercellular communication.^20^ Since 80% of SNPs associated with cancer are located in the non‐coding regions,^21^ many of them are likely to be in lncRNAs.^22^, ^23^ Studies have shown that SNPs in the lncRNA genes can influence cancer through different biological mechanisms. For example, SNPs can affect the expression of their relevant lncRNAs.^24^ Different SNP genotype in *LINC00673* may affect its binding to *miR‐1231*, which alters the miRNA's activity and influences PTPN11 (protein tyrosine phosphatase, non‐receptor type 11) degradation in an allele‐specific manner.^25^ Genetic polymorphisms can also affect the expression of lncRNAs through allele‐specific modulation of their distal regulatory elements. A SNP located in a distal enhancer of lncRNA *PCAT1* (prostate cancer associated transcript 1) alters the binding of its transcription factors ONECUT2 (one cut homeobox 2) and androgen receptor (AR) to the enhancer and *PCAT1* promoter, thereby affecting the expression of *PCAT1* which is involved in the development and progression of prostate cancer.^26^

**Figure 1:**
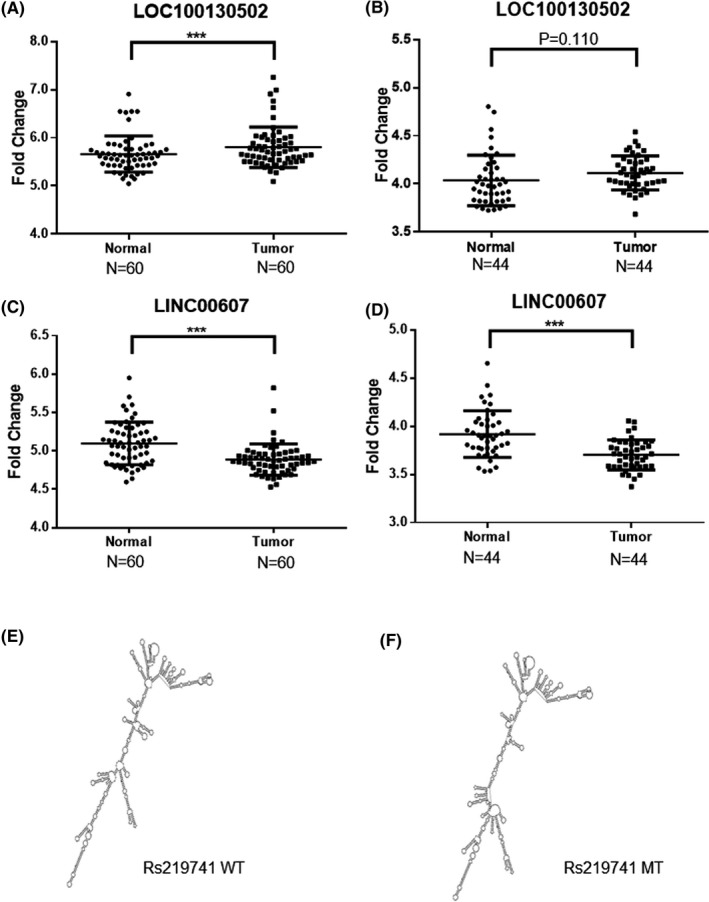
Scatter plots of relative lncRNA levels in NSCLC tumor and adjacent non‐tumor tissues. LOC100130502 in GSE19804 (A) and GSE18842 (B). LINC00607 in GSE19804 (C) and GSE18842 (D). Rs219741 G>A change in lnc‐CHAF1B‐3:1, genotype G (WT) (E), and genotype A (MT) (F). ***P < 0.0001

SNP rs498238 is located in the fourth exon of the long intergenic non‐coding RNA 1833 gene (*LINC01833*), and the lncRNA, initially named as *loc100130502*, is predicted to stay mainly in the nucleus of A549 cells.^27^ In the NCBI GEO database, *loc100130502* was shown to be upregulated in NSCLC tumors compared to matched adjacent non‐tumor tissues of non‐smoking women in one dataset GSE19804 (Figure 1A), but no difference in another dataset GSE18842 (Figure 1B). The *LINC01833* gene is located close to the gene *SIX3*, and this non‐coding transcript is considered a Wnt/β‐catenin pathway‐related lncRNA.^28^ SIX3 was reported to inhibit the pathway in the development of vertebrate forebrain.^29^ Kumar et al^30^ found that SIX3 acted as a corepressor of *Wnt* and suppressed its transcription in breast cancer. In addition, in vivo binding assay revealed that SIX3 repressed Wnt1 expression by binding to its 3′ enhancer and to the elements located within its 5′ promoter region.^31^ SIX3 was downregulated in lung adenocarcinoma tissues compared their matched adjacent normal tissues. Restoration of SIX3 expression in lung cancer cells with low endogenous SIX3 resulted in suppressed cell proliferation and migration. Moreover, high expression of SIX3 was associated with improved overall and progression‐free survival of patients with lung adenocarcinoma.^32^ A similar finding was also observed in patients with glioblastoma.^33^ A meta‐analysis suggests that SIX3 may play a role in suppressing the progression of lung cancer, especially in its early stage.^34^

SNP rs3113503 is an intron variant which is located in a gene encoding two long non‐coding transcripts, including a shorter lncRNA named *LINC01614* and a longer one called *LINC00607*. *LINC00607* is present mainly in cell nucleus,^27^ and significant downregulation was observed in NSCLC when we analyzed the online datasets GSE19804 (Figure 1C) and GSE18842 (Figure 1D). No expression information was found for *lnc‐NDUFS6‐5:5* (rs16901995) and *loc105369301* (rs219741). LncRNASNP database indicates that SNP rs219741 may change the secondary structure of the lncRNA *lnc‐CHAF1B‐3:1* (Figure 1E for wild type and Figure 1F for mutant type). Our data suggest that SNP rs498238 and rs3113503 may have allele‐specific influences on lncRNA expression in NSCLC.

The SNPs we investigated in this study were selected from a list of lncRNAs which showed significant differences in expression between NSCLC tumor and matched adjacent normal tissues. The initial analysis of lncRNAs was accomplished with an expression microarray, and the study population was Chinese Han. Thus, the findings of our SNP analysis were likely to be limited to Chinese populations and the number of lncRNAs included in the microarray chip. In addition to these limitations, our sample size for analyzing the SNP association was relatively small, and there were no validation and *P* value adjustment during our evaluation. We also did not perform any functional evaluation and experiments to demonstrate the biological relevance of these SNPs in NSCLC. Despite these shortcomings, we were able to find some preliminary data to suggest that SNPs in non‐coding regions, especially in the lncRNA genes, may have potential implications in cancer etiology. More studies are needed to characterize these non‐coding region SNPs and elucidate their biological relevance and molecular mechanisms in relation to lncRNA's function and tumorigenesis.

In summary, we analyzed 17 SNPs in the genes of lncRNAs with differential expression in NSCLC and identified three of them associated with the risk of NSCLC among Chinese. These findings suggest that SNPs in non‐coding regions of the genome may also be important when comparing to those in the coding regions. Further analyzing this type of SNPs may provide new insights into the functions of lncRNAs and their involvement in cancer.
