# Supplementary material for: SNPs in LncRNA genes are associated with non‐small cell lung cancer in a Chinese population
Source: J Clin Lab Anal. 2019 Apr 13;33(4):e22858. doi: 10.1002/jcla.22858 (PMC6528608; doi:10.1002/jcla.22858)
Supplement: Supplementary file 1 [file JCLA-33-e22858-s001.docx]

SNPs in LncRNA Genes are Associated with Non-Small Cell Lung Cancer in a Chinese Population.

**Ruoyang Wang ^1^, Nannan Feng ^1^, Yu Wang ^1^, Sumeng Gao ^1^, Fangfang Zhang ^1^, Ying Qian ^1^, Ming Gao ^2^, Herbert Yu ^3^, Baosen Zhou ^4,^ *, Biyun Qian ^1,^ ***

^1^ School of Public Health, Shanghai Jiaotong University School of Medicine, Hongqiao International Institute of Medicine, Shanghai Tongren Hospital, Shanghai, 200025, China.

^2^ Key Laboratory of Cancer Prevention and Therapy, Tianjin Medical University Cancer Institute and Hospital, Tianjin, 300060, China.

^3^ Cancer Epidemiology Program, University of Hawaii Cancer Center, 701 Ilalo Street, Honolulu, HI 96813, USA.

^4^ Department of Epidemiology, School of Public Health, China Medical University, Shenyang 110122, China.

**Supplementary Table S1.** Distribution of the selected characteristics in cases and controls

| Variables | N (%) | | *p*^†^ |
| --- | --- | --- | --- |
|  | Case(n=1294) | Control(n=1729) |  |
| Gender^‡^ | 1171(100%) | 1584(100%) | <0.001 |
| Male | 580(49.53%) | 591(37.31%) |  |
| Female | 591(50.47%) | 993(62.69%) |  |
| Age^‡^ | 1169(100%) | 1354(100%) | <0.001 |
| <60 | 584(49.96%) | 868(64.11%) |  |
| ≥60 | 585(50.04%) | 486(35.89%) |  |
| Smoking status^‡^ | 1171(100%) | 1719(100%) | <0.001 |
| Non-smoker | 610(52.09%) | 1440(83.77%) |  |
| Ever-smoker | 561(47.91%) | 279(16.23%) |  |

^†^Two-side χ^2^ test.

^‡^Due to the missing values, the numbers of cases and controls were less than 1294 and 1729, respectively.

**Supplementary Table S2.** Associations between 17 SNPs in 13 lncRNA genes and NSCLC risk after PSM

| Genotypes | N (%) | | *p*^†^ | Crude OR (95%CI) | Adjusted OR (95%CI) ^‡^ |
| --- | --- | --- | --- | --- | --- |
|  | Case | Control |  |  |  |
| **rs10889184** | 1132(100%) | 986(100%) | 0.836 |  |  |
| GG | 241(21.29%) | 205(20.79%) |  | 1.00 | 1.00 |
| GA | 569(50.27%) | 489(49.59%) |  | 0.99(0.79-1.24) | 0.96 (0.76 -1.21) |
| AA | 322(28.45%) | 292(29.61%) |  | 0.94(0.74-1.20) | 0.91 (0.70 -1.17) |
| Dominant model | 1132(100%) | 986(100%) | 0.779 |  |  |
| GG | 241(21.29%) | 205(20.79%) |  | 1.00 | 1.00 |
| GA+AA | 891(78.71%) | 781(79.21%) |  | 0.97(0.79-1.20) | 0.94 (0.75 -1.17) |
| Recessive model | 1132(100%) | 986(100%) | 0.554 |  |  |
| GG+GA | 810(71.55%) | 694(70.39%) |  | 1.00 | 1.00 |
| AA | 322(28.45%) | 292(29.61%) |  | 0.95(0.78-1.14) | 0.93 (0.76 -1.13) |
| **rs3113503** | 1012(100%) | 979(100%) | 0.050 |  |  |
| GG | 432(42.69%) | 448(45.76%) |  | 1.00 | 1.00 |
| GC | 489(48.32%) | 423(43.21%) |  | 1.20(1.00-1.44) | **1.22 (1.01 -1.49)** |
| CC | 91(8.99%) | 108(11.03%) |  | 0.87(0.64-1.19) | 0.81 (0.59 -1.13) |
| Dominant model | 1012(100%) | 979(100%) | 0.167 |  |  |
| GG | 432(42.69%) | 448(45.76%) |  | 1.00 | 1.00 |
| GC+CC | 580(57.31%) | 531(54.24%) |  | 1.13(0.95-1.35) | 1.14 (0.94 -1.37) |
| Recessive model | 1012(100%) | 979(100%) | 0.129 |  |  |
| GG+GC | 921(91.01%) | 871(88.97%) |  | 1.00 | 1.00 |
| CC | 91(8.99%) | 108(11.03%) |  | 0.80(0.59-1.07) | **0.74 (0.54 -1.00)** |
| **rs498238** | 1005(100%) | 961(100%) | 0.188 |  |  |
| CC | 791(78.71%) | 745(77.52%) |  | 1.00 | 1.00 |
| CT | 209(20.80%) | 204(21.23%) |  | 0.97(0.78-1.20) | 0.97 (0.77 -1.22) |
| TT | 5(0.50%) | 12(1.25%) |  | 0.39(0.14-1.12) | **0.33 (0.11 -0.97)** |
| Dominant model | 1005(100%) | 961(100%) | 0.526 |  |  |
| CC | 791(78.71%) | 745(77.52%) |  | 1.00 | 1.00 |
| CT+TT | 214(21.29%) | 216(22.48%) |  | 0.93(0.75-1.16) | 0.93 (0.74 -1.16) |
| Recessive model | 1005(100%) | 961(100%) | 0.072 |  |  |
| CC+CT | 1000(99.50%) | 949(98.75%) |  | 1.00 | 1.00 |
| TT | 5(0.50%) | 12(1.25%) |  | 0.40(0.14-1.13) | **0.33 (0.11 -0.97)** |
| **rs496467** | 1058(100%) | 981(100%) | 0.399 |  |  |
| AA | 263(24.86%) | 250(25.48%) |  | 1.00 | 1.00 |
| AG | 533(50.38%) | 513(52.29%) |  | 0.99(0.80-1.22) | 1.03 (0.83 -1.29) |
| GG | 262(24.76%) | 218(22.22%) |  | 1.14(0.89-1.47) | 1.14 (0.88 -1.48) |
| Dominant model | 1058(100%) | 981(100%) | 0.745 |  |  |
| AA | 263(24.86%) | 250(25.48%) |  | 1.00 | 1.00 |
| AG+GG | 795(75.14%) | 731(74.52%) |  | 1.03(0.85-1.26) | 1.06 (0.86 -1.31) |
| Recessive model | 1058(100%) | 981(100%) | 0.177 |  |  |
| AA+AG | 796(75.24%) | 763(77.78%) |  | 1.00 | 1.00 |
| GG | 262(24.76%) | 218(22.22%) |  | 1.15(0.94-1.42) | 1.12 (0.90 -1.39) |
| **rs13431201** | 1108(100%) | 1000(100%) | 0.272 |  |  |
| CC | 994(89.71%) | 879(87.90%) |  | 1.00 | 1.00 |
| CG | 111(10.02%) | 115(11.50%) |  | 0.85(0.65-1.13) | 0.82 (0.61 -1.09) |
| GG | 3(0.27%) | 6(0.60%) |  | 0.44(0.11-1.77) | 0.34 (0.08 -1.51) |
| Dominant model | 1108(100%) | 1000(100%) | 0.187 |  |  |
| CC | 994(89.71%) | 879(87.90%) |  | 1.00 | 1.00 |
| CG+GG | 114(10.29%) | 121(12.10%) |  | 0.83(0.64-1.09) | 0.79 (0.59 -1.06) |
| Recessive model | 1108(100%) | 1000(100%) | 0.247 |  |  |
| CC+CG | 1105(99.73%) | 994(99.40%) |  | 1.00 | 1.00 |
| GG | 3(0.27%) | 6(0.60%) |  | 0.45(0.11-1.80) | 0.35 (0.08 -1.54) |
| **rs1992825** | 1086(100%) | 973(100%) | 0.467 |  |  |
| GG | 498(45.86%) | 426(43.78%) |  | 1.00 | 1.00 |
| GC | 483(44.48%) | 459(47.17%) |  | 0.90(0.75-1.08) | 0.91 (0.75 -1.10) |
| CC | 105(9.67%) | 88(9.04%) |  | 1.02(0.75-1.39) | 1.06 (0.77 -1.47) |
| Dominant model | 1086(100%) | 973(100%) | 0.345 |  |  |
| GG | 498(45.86%) | 426(43.78%) |  | 1.00 | 1.00 |
| GC+CC | 588(54.14%) | 547(56.22%) |  | 0.92(0.77-1.09) | 0.94 (0.78 -1.12) |
| Recessive model | 1086(100%) | 973(100%) | 0.627 |  |  |
| GG+GC | 981(90.33%) | 885(90.96%) |  | 1.00 | 1.00 |
| CC | 105(9.67%) | 88(9.04%) |  | 1.08(0.80-1.45) | 1.11 (0.82 -1.52) |
| **rs517055** | 1075(100%) | 987(100%) | 0.204 |  |  |
| AA | 278(25.86%) | 222(22.49%) |  | 1.00 | 1.00 |
| AT | 540(50.23%) | 518(52.48%) |  | 0.83(0.67-1.03) | 0.86 (0.68 -1.07) |
| TT | 257(23.91%) | 247(25.03%) |  | 0.83(0.65-1.07) | 0.83 (0.64 -1.07) |
| Dominant model | 1075(100%) | 987(100%) | 0.075 |  |  |
| AA | 278(25.86%) | 222(22.49%) |  | 1.00 | 1.00 |
| AT+TT | 797(74.14%) | 765(77.51%) |  | 0.83(0.68-1.02) | 0.85 (0.68 -1.05) |
| Recessive model | 1075(100%) | 987(100%) | 0.555 |  |  |
| AA+AT | 818(76.09%) | 740(74.97%) |  | 1.00 | 1.00 |
| TT | 257(23.91%) | 247(25.03%) |  | 0.94(0.77-1.15) | 0.92 (0.74 -1.14) |
| **rs1466099** | 1073(100%) | 983(100%) | 0.204 |  |  |
| GG | 601(56.01%) | 540(54.93%) |  | 1.00 | 1.00 |
| GA | 391(36.44%) | 385(39.17%) |  | 0.91(0.76-1.10) | 0.91 (0.75 -1.11) |
| AA | 81(7.55%) | 58(5.90%) |  | 1.26(0.88-1.79) | 1.31 (0.90 -1.90) |
| Dominant model | 1073(100%) | 983(100%) | 0.623 |  |  |
| GG | 601(56.01%) | 540(54.93%) |  | 1.00 | 1.00 |
| GA+AA | 472(43.99%) | 443(45.07%) |  | 0.96(0.80-1.14) | 0.96 (0.80 -1.16) |
| Recessive model | 1073(100%) | 983(100%) | 0.137 |  |  |
| GG+GA | 992(92.45%) | 925(94.10%) |  | 1.00 | 1.00 |
| AA | 81(7.55%) | 58(5.90%) |  | 1.30(0.92-1.85) | 1.35 (0.94 -1.95) |
| **rs62288095** | 951(100%) | 979(100%) | 0.200 |  |  |
| CC | 744(78.23%) | 782(79.88%) |  | 1.00 | 1.00 |
| CA | 191(20.08%) | 189(19.31%) |  | 1.06(0.85-1.33) | 1.12 (0.88 -1.42) |
| AA | 16(1.68%) | 8(0.82%) |  | 2.10(0.89-4.94) | 2.00 (0.82 -4.89) |
| Dominant model | 951(100%) | 979(100%) | 0.375 |  |  |
| CC | 744(78.23%) | 782(79.88%) |  | 1.00 | 1.00 |
| CA+AA | 207(21.77%) | 197(20.12%) |  | 1.10(0.89-1.38) | 1.16 (0.92 -1.46) |
| Recessive model | 951(100%) | 979(100%) | 0.086 |  |  |
| CC+CA | 935(98.32%) | 971(99.18%) |  | 1.00 | 1.00 |
| AA | 16(1.68%) | 8(0.82%) |  | 2.08(0.89-4.88) | 1.95 (0.80 -4.78) |
| **rs6830064** | 1095(100%) | 966(100%) | 0.980 |  |  |
| TT | 758(69.22%) | 672(69.57%) |  | 1.00 | 1.00 |
| TG | 304(27.76%) | 266(27.54%) |  | 1.01(0.83-1.23) | 0.96 (0.78 -1.18) |
| GG | 33(3.01%) | 28(2.90%) |  | 1.05(0.63-1.75) | 1.12 (0.65 -1.92) |
| Dominant model | 1095(100%) | 966(100%) | 0.867 |  |  |
| TT | 758(69.22%) | 672(69.57%) |  | 1.00 | 1.00 |
| TG+GG | 337(30.78%) | 294(30.43%) |  | 1.02(0.84-1.23) | 0.97 (0.80 -1.18) |
| Recessive model | 1095(100%) | 966(100%) | 0.878 |  |  |
| TT+TG | 1062(96.99%) | 938(97.10%) |  | 1.00 | 1.00 |
| GG | 33(3.01%) | 28(2.90%) |  | 1.04(0.62-1.74) | 1.13 (0.66 -1.94) |
| **rs7678341** | 1082(100%) | 980(100%) | 0.799 |  |  |
| GG | 640(59.15%) | 590(60.20%) |  | 1.00 | 1.00 |
| GA | 377(34.84%) | 337(34.39%) |  | 1.03(0.86-1.24) | 1.04 (0.86 -1.26) |
| AA | 65(6.01%) | 53(5.41%) |  | 1.13(0.77-1.65) | 1.05 (0.70 -1.56) |
| Dominant model | 1082(100%) | 980(100%) | 0.626 |  |  |
| GG | 640(59.15%) | 590(60.20%) |  | 1.00 | 1.00 |
| GA+AA | 442(40.85%) | 390(39.80%) |  | 1.05(0.88-1.25) | 1.04 (0.87 -1.25) |
| Recessive model | 1082(100%) | 980(100%) | 0.559 |  |  |
| GG+GA | 1017(93.99%) | 927(94.59%) |  | 1.00 | 1.00 |
| AA | 65(6.01%) | 53(5.41%) |  | 1.12(0.77-1.62) | 1.03 (0.70 -1.53) |
| **rs16901995** | 1096(100%) | 984(100%) | 0.413 |  |  |
| CC | 380(34.67%) | 322(32.72%) |  | 1.00 | 1.00 |
| CT | 532(48.54%) | 477(48.48%) |  | 0.95(0.78-1.15) | 0.94 (0.77 -1.15) |
| TT | 184(16.79%) | 185(18.80%) |  | 0.84(0.66-1.09) | 0.78 (0.59 -1.01) |
| Dominant model | 1096(100%) | 984(100%) | 0.348 |  |  |
| CC | 380(34.67%) | 322(32.72%) |  | 1.00 | 1.00 |
| CT+TT | 716(65.33%) | 662(67.28%) |  | 0.92(0.76-1.10) | 0.89 (0.74 -1.08) |
| Recessive model | 1096(100%) | 984(100%) | 0.230 |  |  |
| CC+CT | 912(83.21%) | 799(81.20%) |  | 1.00 | 1.00 |
| TT | 184(16.79%) | 185(18.80%) |  | 0.87(0.70-1.09) | 0.80 (0.63 -1.02) |
| **rs4077205** | 1094(100%) | 989(100%) | 0.153 |  |  |
| AA | 108(9.87%) | 107(10.82%) |  | 1.00 | 1.00 |
| AG | 476(43.51%) | 389(39.33%) |  | 1.21(0.90-1.64) | 1.29 (0.94 -1.77) |
| GG | 510(46.62%) | 493(49.85%) |  | 1.03(0.76-1.38) | 1.11 (0.81 -1.51) |
| Dominant model | 1094(100%) | 989(100%) | 0.478 |  |  |
| AA | 108(9.87%) | 107(10.82%) |  | 1.00 | 1.00 |
| AG+GG | 986(90.13%) | 882(89.18%) |  | 1.11(0.84-1.47) | 1.19 (0.88 -1.60) |
| Recessive model | 1094(100%) | 989(100%) | 0.141 |  |  |
| AA+AG | 584(53.38%) | 496(50.15%) |  | 1.00 | 1.00 |
| GG | 510(46.62%) | 493(49.85%) |  | 0.88(0.74-1.04) | 0.90 (0.75 -1.08) |
| **rs35132843** | 1044(100%) | 972(100%) | 0.242 |  |  |
| TT | 389(37.26%) | 381(39.20%) |  | 1.00 | 1.00 |
| TG | 500(47.89%) | 471(48.46%) |  | 1.04(0.86-1.26) | 1.03 (0.84 -1.25) |
| GG | 155(14.85%) | 120(12.35%) |  | 1.27(0.96-1.67) | 1.19 (0.89 -1.59) |
| Dominant model | 1044(100%) | 972(100%) | 0.371 |  |  |
| TT | 389(37.26%) | 381(39.20%) |  | 1.00 | 1.00 |
| TG+GG | 655(62.74%) | 591(60.80%) |  | 1.09(0.91-1.30) | 1.06 (0.88 -1.28) |
| Recessive model | 1044(100%) | 972(100%) | 0.102 |  |  |
| TT+TG | 889(85.15%) | 852(87.65%) |  | 1.00 | 1.00 |
| GG | 155(14.85%) | 120(12.35%) |  | 1.24(0.96-1.60) | 1.17 (0.90 -1.53) |
| **rs10734387** | 1062(100%) | 990(100%) | 0.561 |  |  |
| CC | 95(8.95%) | 80(8.08%) |  | 1.00 | 1.00 |
| CT | 443(41.71%) | 434(43.84%) |  | 0.86(0.62-1.19) | 0.78 (0.55 -1.09) |
| TT | 524(49.34%) | 476(48.08%) |  | 0.93(0.67-1.28) | 0.82 (0.59 -1.16) |
| Dominant model | 1062(100%) | 990(100%) | 0.484 |  |  |
| CC | 95(8.95%) | 80(8.08%) |  | 1.00 | 1.00 |
| CT+TT | 967(91.05%) | 910(91.92%) |  | 0.90(0.66-1.22) | 0.80 (0.58 -1.11) |
| Recessive model | 1062(100%) | 990(100%) | 0.568 |  |  |
| CC+CT | 538(50.66%) | 514(51.92%) |  | 1.00 | 1.00 |
| TT | 524(49.34%) | 476(48.08%) |  | 1.05(0.88-1.25) | 1.02 (0.85 -1.22) |
| **rs1867299** | 1101(100%) | 988(100%) | 0.181 |  |  |
| TT | 35(3.18%) | 40(4.05%) |  | 1.00 | 1.00 |
| TC | 318(28.88%) | 254(25.71%) |  | 1.43(0.88-2.32) | 1.38 (0.83 -2.29) |
| CC | 748(67.94%) | 694(70.24%) |  | 1.23(0.77-1.96) | 1.18 (0.72 -1.91) |
| Dominant model | 1101(100%) | 988(100%) | 0.286 |  |  |
| TT | 35(3.18%) | 40(4.05%) |  | 1.00 | 1.00 |
| TC+CC | 1066(96.82%) | 948(95.95%) |  | 1.29(0.81-2.04) | 1.23 (0.76 -1.99) |
| Recessive model | 1101(100%) | 988(100%) | 0.255 |  |  |
| TT+TC | 353(32.06%) | 294(29.76%) |  | 1.00 | 1.00 |
| CC | 748(67.94%) | 694(70.24%) |  | 0.90(0.75-1.08) | 0.88 (0.73 -1.08) |
| **rs219741** | 1056(100%) | 955(100%) | 0.325 |  |  |
| GG | 813(76.99%) | 753(78.85%) |  | 1.00 | 1.00 |
| GA | 235(22.25%) | 191(20.00%) |  | 1.14(0.92-1.41) | 1.08 (0.86 -1.35) |
| AA | 8(0.76%) | 11(1.15%) |  | 0.67(0.27-1.68) | 0.60 (0.23 -1.56) |
| Dominant model | 1056(100%) | 955(100%) | 0.316 |  |  |
| GG | 813(76.99%) | 753(78.85%) |  | 1.00 | 1.00 |
| GA+AA | 243(23.01%) | 202(21.15%) |  | 1.11(0.90-1.38) | 1.05 (0.84 -1.31) |
| Recessive model | 1056(100%) | 955(100%) | 0.361 |  |  |
| GG+GA | 1048(99.24%) | 944(98.85%) |  | 1.00 | 1.00 |
| AA | 8(0.76%) | 11(1.15%) |  | 0.66(0.26-1.64) | 0.59 (0.23 -1.53) |

Bold OR values indicated *p*<0.05.

^†^Two-side χ2 test.

^‡^Adjusted for age, gender, and smoking status.

**Supplementary Table S3.** Clinical and pathological characteristics of NSCLC patients

| Variables | Patients | Deaths | MST (months)(95%CI) | Log-rank *p* | HR (95%CI) |
| --- | --- | --- | --- | --- | --- |
| Gender | 869 | 527 |  | **<0.001** |  |
| Male | 432 | 216 | 60.00(46.80-73.20) |  | 1.00 |
| Female | 437 | 311 | 24.53(21.55-27.52) |  | **1.87(1.57-2.22)** |
| Age | 868 | 527 |  | 0.522 |  |
| <60 | 442 | 265 | 32.33(27.67-36.99) |  | 1.00 |
| ≥60 | 426 | 262 | 29.27(23.81-34.72) |  | 1.06(0.89-1.25) |
| Smoking status | 867 | 527 |  | **<0.001** |  |
| Non-smoker | 414 | 302 | 23.60(20.49-26.71) |  | 1.00 |
| Ever-smoker | 453 | 225 | 60.70(48.12-73.28) |  | **0.50(0.42-0.60)** |
| Disease stage | 780 | 490 |  | **<0.001** |  |
| Ⅰ/Ⅱ | 392 | 189 | 68.23(60.13-76.34) |  | 1.00 |
| Ⅲ/Ⅳ | 388 | 301 | 20.73(17.86-23.60) |  | **2.47(2.06-2.97)** |
| Histology type^†^ | 854 | 516 |  | **0.002** |  |
| ADC | 460 | 289 | 26.53(23.34-29.72) |  | 1.00 |
| SCC | 355 | 197 | 47.60(34.12-61.08) |  | **0.74(0.61-0.88)** |
| ASC | 35 | 28 | 34.70(12.96-56.44) |  | 1.27(0.86-1.88) |
| LCLC | 4 | 2 | 30.98(10.02-51.93) |  | 1.10(0.27-4.41) |

Bold HR values indicated *p*<0.05.

^†^ADC (Adenocarcinomas); SCC (Squamous cell carcinomas); ASC (Adeno-Squamous Carcinoma); LCLC (Large cell lung cancer).

**Supplementary Table S4.** Associations between SNPs and NSCLC survival stratified by clinicopathological variables

| Genetic Variant | Variables | Genotypes (Deaths/Patients) | | *p* value ^†^ | Dominant model (AB + BB)/AA HR (95 % CI) ^†^ |
| --- | --- | --- | --- | --- | --- |
|  |  | AA^‡^ | AB+BB^‡^ |  |  |
| rs3113503 | Gender |  |  |  |  |
|  | Male | 94/179 | 98/201 | 0.884 | 0.98(0.72-1.33) |
|  | Female | 104/147 | 161/218 | 0.352 | 1.13(0.87-1.47) |
|  | Age |  |  |  |  |
|  | <60 | 102/166 | 124/207 | 0.427 | 1.12(0.85-1.48) |
|  | ≥60 | 96/160 | 135/212 | 0.849 | 1.03(0.77-1.37) |
|  | Smoking status |  |  |  |  |
|  | Non-smoker | 109/149 | 151/200 | 0.549 | 1.08(0.83-1.41) |
|  | Ever-smoker | 89/177 | 108/217 | 0.542 | 1.10(0.81-1.49) |
|  | Disease stage |  |  |  |  |
|  | Ⅰ/Ⅱ | 72/138 | 91/196 | 0.689 | 0.94(0.68-1.29) |
|  | Ⅲ/Ⅳ | 109/148 | 152/185 | 0.128 | 1.22(0.94-1.58) |
|  | Histology type* |  |  |  |  |
|  | ADC | 99/168 | 150/224 | 0.103 | 1.25(0.96-1.64) |
|  | SCC | 79/134 | 91/171 | 0.213 | 0.82(0.60-1.12) |
|  | ASC | 14/16 | 11/13 | 0.079 | 0.37(0.12-1.12) |
|  | LCLC | 1/2 | 1/2 | - | - |
| rs498238 | Gender |  |  |  |  |
|  | Male | 140/283 | 42/90 | 0.274 | 0.81(0.56-1.18) |
|  | Female | 213/290 | 53/73 | 0.495 | 1.12(0.82-1.53) |
|  | Age |  |  |  |  |
|  | <60 | 173/286 | 48/83 | 0.839 | 1.04(0.74-1.45) |
|  | ≥60 | 180/287 | 47/80 | 0.680 | 0.93(0.65-1.32) |
|  | Smoking status |  |  |  |  |
|  | Non-smoker | 207/271 | 50/74 | 0.919 | 0.98(0.71-1.36) |
|  | Ever-smoker | 146/301 | 45/88 | 0.813 | 0.96(0.66-1.38) |
|  | Disease stage |  |  |  |  |
|  | Ⅰ/Ⅱ | 130/262 | 33/70 | 0.871 | 0.97(0.66-1.43) |
|  | Ⅲ/Ⅳ | 199/251 | 54/74 | 0.754 | 0.95(0.70-1.30) |
|  | Histology type* |  |  |  |  |
|  | ADC | 196/299 | 50/89 | 0.875 | 0.97(0.70-1.35) |
|  | SCC | 127/238 | 41/66 | 0.969 | 1.01(0.69-1.47) |
|  | ASC | 19/21 | 3/6 | 0.853 | 1.14(0.30-4.37) |
|  | LCLC | 2/4 | 0/0 | - | - |
| rs16901995 | Gender |  |  |  |  |
|  | Male | 66/135 | 136/269 | 0.250 | 0.83(0.61-1.14) |
|  | Female | 116/158 | 181/247 | 0.507 | 1.09(0.85-1.39) |
|  | Age |  |  |  |  |
|  | <60 | 97/155 | 152/252 | 0.163 | 0.83(0.63-1.08) |
|  | ≥60 | 85/138 | 165/264 | 0.565 | 1.09(0.82-1.44) |
|  | Smoking status |  |  |  |  |
|  | Non-smoker | 115/157 | 172/224 | 0.370 | 1.12(0.88-1.43) |
|  | Ever-smoker | 67/135 | 145/291 | 0.510 | 0.90(0.66-1.23) |
|  | Disease stage |  |  |  |  |
|  | Ⅰ/Ⅱ | 70/137 | 109/227 | 0.924 | 0.99(0.72-1.35) |
|  | Ⅲ/Ⅳ | 100/131 | 186/232 | 0.823 | 0.97(0.76-1.25) |
|  | Histology type* |  |  |  |  |
|  | ADC | 109/164 | 170/269 | 0.575 | 1.08(0.83-1.39) |
|  | SCC | 63/114 | 119/212 | 0.524 | 0.90(0.65-1.24) |
|  | ASC | 8/11 | 18/21 | 0.306 | 0.59(0.22-1.61) |
|  | LCLC | 1/1 | 1/3 | - | - |
| rs219741 | Gender |  |  |  |  |
|  | Male | 140/305 | 53/91 | 0.462 | 1.14(0.81-1.61) |
|  | Female | 219/304 | 55/76 | 0.791 | 1.04(0.76-1.42) |
|  | Age |  |  |  |  |
|  | <60 | 182/310 | 52/82 | 0.675 | 1.07(0.78-1.48) |
|  | ≥60 | 177/299 | 56/85 | 0.389 | 1.15(0.83-1.59) |
|  | Smoking status |  |  |  |  |
|  | Non-smoker | 210/284 | 55/75 | 0.878 | 1.03(0.75-1.40) |
|  | Ever-smoker | 149/323 | 53/92 | 0.323 | 1.18(0.85-1.65) |
|  | Disease stage |  |  |  |  |
|  | Ⅰ/Ⅱ | 125/277 | 41/71 | 0.058 | 1.42(0.99-2.02) |
|  | Ⅲ/Ⅳ | 207/267 | 60/80 | 0.628 | 0.93(0.69-1.25) |
|  | Histology type* |  |  |  |  |
|  | ADC | 197/319 | 59/89 | 0.286 | 1.18(0.87-1.59) |
|  | SCC | 131/250 | 43/70 | 0.362 | 1.19(0.82-1.71) |
|  | ASC | 19/23 | 5/6 | **0.035** | **0.22(0.05-0.90)** |
|  | LCLC | 2/4 | 0/0 | - | - |

Bold HR values indicated *p*<0.05.

^†^Adjusted for age, gender, smoking status, disease stage, and histology type when properly.

^‡^AA – wild genotype; BB – variant genotype; AB – heterogenous.

* ADC (Adenocarcinomas); SCC (Squamous cell carcinomas); ASC (Adeno-Squamous Carcinoma); LCLC (Large cell lung cancer).
